# It Takes a Village!

**DOI:** 10.1016/j.artd.2025.101781

**Published:** 2025-07-25

**Authors:** Brett R. Levine

Over the past 7 months, since my appointment as the editor-in-chief of Arthroplasty Today, I have come to the realization that the success of this publication is heavily reliant on the individuals who support it. The editorial staff at Elsevier and Drs. Cohen-Rosenblum and Leiberman have been invaluable in facilitating this process. A special thanks to Megan Griffiths for all of your assistance and guidance, and welcome Stephanie Turner to the team (you have big shoes to fill!). To accommodate the growing volume of submissions, we have implemented significant changes to our associate editor roster. Our current associate editors include Stefano Bini, Thomas Blumenfeld, Jonathan Danoff, Daniel Kendoff, Rina Jain, Adam Edelstein, Nick Brown, Eli Kamara, Aiden Pour, and Ayesha Abdeen. Their thoughtful insights have significantly influenced the articles that are accepted for publication in Arthroplasty Today. As a result of these efforts, our 2024 impact factor has been upgraded to 2.1, an increase from 1.5 in 2023.

Our editorial board has also seen significant changes as we have had many members that were on the original board and have completed their service to the journal. I would like to thank Drs. Sonny Bal, David Blaha, Michael Bolognesi, James Browne, Craig Della Valle, Anthony Unger, Gurava Reddy, Thiago Busato, Jason Davis, Ronald Delanois, and Adam Schwartz. Your contributions to Arthroplasty Today have been outstanding and we would not be in the position we are today without you all. I would also like to welcome new members of the editorial board, including, Drs. Meghan Whitmarsh-Brown, Kevin Sonn, Chris Melnic, Aldo Resiego, Praharsha Mulpur, Anderson Freitas, James Nace, and Katharine Harper. The remainder of the Editorial Board that is staying on for an additional 2-year term include: Drs. Elizabeth Dailey, Matthew Bullock, Adam Edelstein, Ashton Goldman, S. Hammouche, Joshua Rainey, David Markel, J. Bo Mason, Hari Parvateneni, Krishna Tripuraneni, Frank DiMaio, Nathaniel Nelms, Ben Ricciardi, Lee Rubin, Ran Schwarzkopf, Jason Wesstein, and Erik Zeegan. Once again thank for all of your contributions as it truly takes a village to succeed. For all of our authors and researchers, keep the submissions coming we have a full staff to handle the workload and promise to keep adding more as we grow.

In this editorial, amid the challenging times in health care, politics, and the global arena, I will focus on the field of orthopaedics. I trust that our readers are finding the thought-provoking guest editorials and the informative “Did You Know” articles from the Evidence-Based Medicine Committee to be engaging. Some of the highlights from the original research articles in Volume 34 include: a clinical research paper on targeting opioid reduction in primary total hip arthroplasty (with a goal to go opioid-free), use of an artificial intelligence–powered Chatbot to improve clinical outcomes and satisfaction after periacetabular osteotomy surgery and a systematic review looking at the association between cup positioning and outcomes for patients with iliopsoas impingement syndrome.

Additionally, there is a case report describing the use of mesh reconstruction for nonunion of a patella fracture in the setting of osteoarthritis. There is also an excellent surgical technique manuscript covering robotic assisted conversion of a unicompartmental knee to a total knee arthroplasty. These are just a few of the highlights as I encourage you to peruse the issue in its entirety.

Stay tuned, as in upcoming issues there will be another guest editorial, resident/student article on what makes an outstanding Adult Reconstruction away rotation and an article/video on guidance with putting meeting presentations together. I truly appreciate the support of our Presidential Line, Dr. Mont and all of our readers. With our village in hand we will keep growing and furthering the American Association of Hip and Knee Surgeons mission to excel in advancing excellence in hip and knee care.

Respectfully,

Brett R. Levine, MD, MS

Editor-in-Chief

## Conflicts of interest

The author declares there are no conflicts of interest.

For full disclosure statements refer to https://doi.org/10.1016/j.artd.2025.101781.

